# Preoperative communication between anaesthetists and patients with obesity regarding perioperative risks and weight management: a structured narrative review

**DOI:** 10.1186/s13741-020-00154-4

**Published:** 2020-08-13

**Authors:** Anthony Hodsdon, Natalie Anne Smith, David A. Story

**Affiliations:** 1 Department of Anaesthetics, Wollongong Hospital, Loftus St., Wollongong, NSW 2500 Australia; 2 Centre for Integrated Critical Care, Department of Medicine & Radiology, Melbourne Medical School, Faculty of Medicine, Dentistry & Health Sciences, The University of Melbourne, 151 Barry Street, Parkville, VIC 3010 Australia

**Keywords:** Perioperative medicine, Anaesthesia, Obesity, Counselling

## Abstract

**Background:**

Individuals with obesity frequently present for anaesthesia and surgery. Good communication during the preoperative consultation can optimise the provision of relevant health information and guide improvement of health status preoperatively.

**Methods:**

We planned a systematic literature review to assess existing guidelines and evidence of effectiveness for how anaesthetists should communicate with patients who have obesity in the preoperative period about perioperative risks and weight management. Database searches used keywords related to perioperative weight loss conversations. We found no papers that directly addressed our aim. The literature identified as most relevant was analysed in the form of a narrative review.

**Results:**

The majority of suggestions for weight loss conversations came from primary care. Four primary themes potentially relevant to anaesthetists were identified: barriers to such conversations, communication tools, language and communication and specific recommendations. Identified barriers included lack of skills, training, poor remuneration, pessimism and time constraints for clinicians. Established discussion tools including the ‘5A’s’ approach (Assess, Advise, Agree, Assist, Arrange) and motivational interviewing may hold promise to improve preoperative conversations. The papers highlighted a need for empathetic language, including use of patient-specific language where possible.

**Conclusions:**

There are currently no published guidelines for how anaesthetists could most effectively discuss weight in the perioperative period with patients who have obesity. Much of the literature for obesity communication is based on the primary care setting. The perioperative period may represent an increased time of receptiveness for patients. Guidelines for discussions about weight management and associated perioperative risk are suggested.

## Background

Obesity is an increasingly common problem in many countries (Twells et al., 2014) with many potential health implications in the perioperative period (Nightingale et al., 2015). Rates of obesity in surgical patients have been reported to vary between 35 and 70% depending on the type of surgery and can be twice the background rate of the general population (Mullen et al., 2009; AIHW, 2019; STARSurg-Collaborative, 2016; Hamlin et al., 2013; Freckelton et al., 2019; Harms et al., 2007). Preoperative problems include optimisation of concurrent medical conditions such as obstructive sleep apnoea (Chung et al., 1999; Abdullah & Chung, 2014). Intraoperative concerns include mechanical problems such as accurate blood pressure measurement, intravenous access and adequacy of ventilation (Chung et al., 1999; Abdullah & Chung, 2014). Postoperative complications include respiratory failure requiring prolonged endotracheal intubation and intensive care, myocardial infarction and cardiac arrest, wound infection, urinary tract infection, pulmonary embolism, renal failure, peripheral nerve injury and prolonged time in the post-anaesthesia care unit (PACU) with respiratory difficulties (Chung et al., 1999; Bamgbade et al., 2007; Chen et al., 2011; Merkow et al., 2009; Blouw et al., 2003).

Anaesthetists face many unique communication challenges with patients (Hool & Smith, 2009; Kopp & Shafer, 2000). Their contact together is usually brief and often time-pressured. Patients may be anxious, in pain, acutely unwell or affected by medications such as potent analgesics. The perioperative period is in itself a time of vulnerability for patients (Cousley, 2015). They are dealing with a health problem that may vary in severity and urgency, with uncertain outcomes, admission to hospital, multiple health care providers and the loss of control that occurs with anaesthesia and surgery. The necessity for a surgical procedure may be related to lifestyle factors such as smoking and obesity. All of these factors support the need for high-quality professional communication skills, which are rarely explicitly taught (Hool & Smith, 2009; Kopp & Shafer, 2000).

Preoperative consultation with an anaesthetist generally occurs either days or weeks before the day of surgery in a preoperative assessment clinic (PAC), or immediately prior to surgery on the day of admission. Both situations offer only a limited opportunity to address significant behaviour change prior to the planned procedure. The anaesthetist would ideally cover two related but separate issues with patients who have obesity: the risks associated with obesity in the perioperative period, and encouragement for weight loss (Nightingale et al., 2015; Chung et al., 1999; Abdullah & Chung, 2014; Bamgbade et al., 2007; Chen et al., 2011; Merkow et al., 2009; Blouw et al., 2003).

As the issue of specific communication between anaesthetists and patients with obesity has been rarely considered, a survey was performed by the Australian and New Zealand College of Anaesthetists (ANZCA) to uncover attitudes and practices related to perioperative communication with these patients. The results of this survey showed that many anaesthetists find it difficult to communicate with patients with obesity about their weight (Hinks, 2015). Approximately two-thirds of the 800 respondents indicated that obesity was the most common co-morbid condition they encounter and the same proportion noted that they had anaesthetised at least one obese patient on their most recent clinical day. The survey found almost universal agreement that obesity increases both perioperative and lifetime risks for patients. However, respondents indicated uncertainty in knowing how best to approach the problem, with concerns about not wanting to cause hurt or offence with chosen language, changing societal norms regarding the increasing prevalence and normalisation of obesity and low patient health literacy regarding obesity and its implications for anaesthesia and surgery.

The lack of specific advice available for anaesthetists with regards to weight loss counselling represents a gap in current literature. Anaesthetists desire guidance on this important and frequent presentation; however, there are no recommended guidelines available. Our aim was to perform a literature review to uncover guidelines as to how anaesthetists should most effectively conduct preoperative conversations with obese patients to include both perioperative risks and weight loss management.

## Methods

The methodology was planned as a systematic literature review. The question for the review was: How can anaesthetists best conduct preoperative conversations regarding perioperative risk and weight loss with patients who have obesity? Formal searches of Ovid MEDLINE® were performed in February 2016 by professional librarians from two institutions (ANZCA and the Illawarra Shoalhaven Local Health District). After finding no relevant literature from the original searches, we repeated the search using expanded search terms in May 2016 (see Table 1 below). Searches were restricted to the English language and covered the period from 2006 to May 2016. The grey literature was not included. Search terms were used both individually and in combination.

**Table 1:** Search terms for literature review

	Key search terms	Additional search terms
Search 1 (February 2016)	Health education/promotion Communication Perioperative care/period Interpersonal/physician-patient relations Obesity/body mass index Weight/weight loss Diet Referral and consultation Narration Anaesth*/anesth*	
Search 2 (May 2016)		Anaesthetist/anaesthesiologist Weight management Directive counselling/Counselling

No studies directly addressing the study question were found. Therefore, a narrative review of the closest related papers was performed based on thematic analysis associated with the research question. We reviewed papers that dealt with the fields of communication between anaesthetists and patients in any situation, between surgeons and patients relating to obesity in any way, any type of perioperative communication and weight loss or management conversations between any health professionals and patients. Literature regarding other patient counselling circumstances such as smoking cessation and drug addiction was also considered if thought to be potentially relevant to a preoperative consultation situation. Abstracts and references from relevant papers were manually searched and papers were added to the evaluation list if they were deemed likely to add value to the study question. This search was conducted by one investigator (AH) and clarified in discussion with a second investigator (NAS) until agreement on papers to be included was reached. Literature regarding other patient counselling circumstances such as smoking cessation and drug addiction was also considered if thought to be potentially relevant to a preoperative consultation situation.

A thematic analysis approach was used. After reviewing the literature base broadly, eight draft themes were agreed upon by the authors. Review was undertaken of each full text paper categorising and coding the content that related to these themes in Microsoft Excel. Following initial review, it was agreed by the authors to collapse the number of themes to four, with the inclusion of subthemes. An obesity physician and a medical communication expert independently reviewed the final collection of themes and no changes were made. Papers were included in the review if they contributed to the selected themes. Due to the varied source and nature of the reviewed publications, and the exploratory nature of the investigation, we did not undertake a formal critical evaluation of the quality of evidence.

## Results

**Fig. 1:**
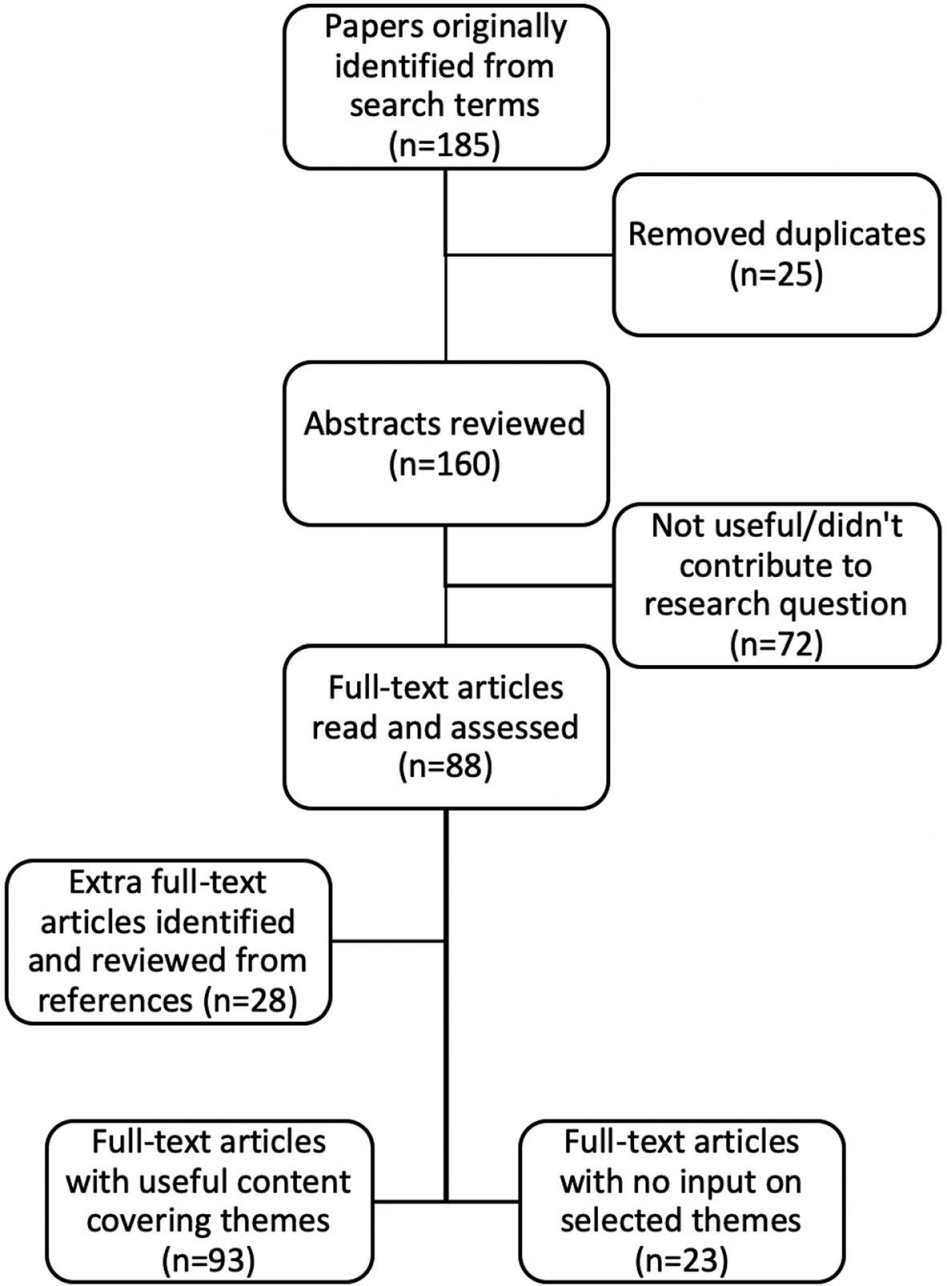
Search method

One hundred and eighty-five papers were originally identified. A total of 116 papers were selected for review, with 95 papers able to provide input to the research question (Fig. 1). These 93 papers came from diverse healthcare backgrounds including primary care (49), communication (18), paediatrics (seven), anaesthesia and perioperative management (six), internal medicine (five), obstetrics (four), dietetics (two), surgical management (one) and allied health (one). The majority of papers relating to weight loss discussions were drawn from primary care and communication literature, with the anaesthesia and perioperative medicine papers relating to general pre-anaesthetic communication rather than specifics of weight counselling in these areas.

The four final themes with subthemes are presented in Table 2. More than one theme was present in most papers. The most common theme related to the barriers to conversations between patients with obesity and health care providers, with 59 of the 93 papers (63%) making reference to barriers. Lack of training (in 45%) and the perception of insufficient time (in 44%) within a consultation were the most commonly found barrier sub-themes.

**Table 2:** Weight loss conversation themes and sub-themes

Barriers to conversations	Communication tools	Language and communication	Specific recommendations
- Lack of training - Insufficient time - Pessimism - Poor resources - Complex topic - More immediate needs	- Motivational interviewing - 5 A’s (Assess, Advise, Agree, Assist, Arrange) - Written materials - 4 E’s (engage, empathise, educate, enlist)	- Specific terminology - Empathetic - Patient-centred (specific)	- Training for physicians - Clear referral pathways - Specific consultation suggestions

Motivational interviewing (in 20%) and the 5 A’s (Assess, Advise, Agree, Assist, Arrange) (in 18%) were the most commonly identified communication tools, with less frequent references to a similar tool, the 4 E’s (engage, empathise, educate, enlist), and written materials. Multiple papers made reference to the terminology recommended when engaging in weight loss conversations. ‘Weight’ was the preferred term for conversations, with ‘fatness’ the least preferred term (Dutton et al., 2010). Patients desired an empathetic approach from their doctor, with patient-centred communication considered of higher value than generic weight loss advice (Huang et al., 2004).

The most common subtheme regarding specific recommendations for weight loss conversations was for physicians to be specifically trained in how to discuss weight with patients with obesity (in 19%). Availability of clear referral pathways to manage obesity was also mentioned (in 10%): this ties in with the ‘assist’ and ‘arrange’ elements of the 5 A’s.

Table 3 provides more detailed information on the papers’ specific contributions to the themes. Although 93 papers provided information, many of these papers contained repeated thematic material; therefore, only the most relevant have been included to provide a summary. Fifty-two quotations from 36 papers are presented in Table 3 that were deemed by the authors to be representative of the themes and subthemes associated with weight loss conversations. This is not an exhaustive list of all thematic data collected during the analysis of the literature; however, the table represents the most salient points relating to our research question.

**Table 3:** Detailed thematic analysis

Theme	Subtheme	Summary	Quotes
Barriers to conversations	Lack of training	Lack of training in weight loss counselling was a significant recurrent barrier in literature	‘Primary care physicians often feel inadequately prepared to provide this counselling… brief weight loss counselling training for resident and primary care physicians may be necessary’ (Huang et al., 2004). ‘Although physicians acknowledge the importance of weight management, many report they lack training and confidence to provide counseling to enhance weight loss’ (Davis et al., 2008). ‘Health professionals repeatedly report a lack of confidence in knowing how to address obesity in their patients. They report minimal, if any, training on obesity as well as limited resources for effective conversations and insufficient clinical time to be able to do this well’ (Gordon & Black, 2016).
	Insufficient time	Lack of time to provide weight loss advice, particularly when there are other issues to address	‘It is likely that GPs have little time during a single consultation to dedicate to lifestyle advice in one consultation’ (Booth & Nowson, 2010). ‘…many physicians report they lack adequate training and time to counsel patients about weight loss and are often fatalistic about the efficacy of obesity treatment’ (Davis et al., 2008). ‘Many providers feel they cannot devote clinical time to weight management when faced with acute and chronic demands to manage disease states and illnesses stemming from diabetes, heart disease, hypertension, and dyslipidemias’ (Greiner et al., 2008).
	Poor resources	Insufficient reimbursement and insufficient resources available to perform weight loss counselling	‘Lack of physical and human resources to encourage and support weight loss was the main perceived barrier to helping patients achieve physical activity and weight loss goals’ (Eley & Eley, 2009). ‘Guidelines may not be specific or operational enough’ (Cox et al., 2011). Multiple papers refer to lack of reimbursement as a barrier to weight loss discussions (Nawaz et al., 1999; Rose et al., 2013; Shiffman et al., 2009).
	Pessimism	Pessimism in regards to patients’ willingness to take on advice/willingness to change/effectiveness of weight loss conversations in general	‘Physicians did not believe that patients would attempt and succeed at losing weight as a result of their counselling’ (Alexander et al., 2007). ‘…physicians tend to have a more negative outlook on patient weight and behaviors than patients do. For example, physicians were more likely than patients to think that patients lack self-control to stay on a diet. Physicians also tend to believe that patients have less motivation and think that patients will lose less weight than patients themselves believe’ (Post et al., 2011).
	Complex topic	Associated with lack of training, physicians felt that the multifactorial nature of obesity makes it difficult to address	‘For diet the situation is even more complex. Sustained reductions in body weight will usually require a cut in energy intake, but responsible dietary management of obesity must also reduce the associated health risks … there is a diverse collection of messages about the overall composition and nutritional balance of the diet, which can be difficult for consumers to assimilate’ (Jebb et al., 2003). ‘…physicians may feel reluctant to address the topic of obesity in the first place, with the fear of offending patients or confirming feelings of failure at weight loss’ (Sherson et al., 2014).
	More immediate needs	Associated to insufficient time, physicians noted that patients often had more acute medical issues to address	‘Many providers feel they cannot devote clinical time to weight management when faced with acute and chronic demands to manage disease states and illnesses stemming from diabetes, heart disease, hypertension, and dyslipidemias’ (Greiner et al., 2008).
Communication tools	5 A’s	Framework: Ask, Advise, Assess, Assist, Arrange Framework for addressing change behaviour, initially developed for smoking cessation. Evidence for effectiveness in weight loss conversations.	‘The 5A’s is a framework developed for smoking cessation counseling in the office setting, and is also useful for most areas of preventive counseling in primary care’ (Jay et al., 2010b). ‘Using the 5A’s … as a weight counseling strategy has been associated with increased motivation to lose weight and increased weight loss’ (Gudzune et al., 2012). ‘The 5As counseling framework is an evidence-based way to teach physicians to counsel obese patients and measure the quality of obesity counseling. This framework guides providers to assess risk, current behavior, and readiness to change, advise change of specific behaviors, agree and collaboratively set goals, assist in addressing barriers and securing support, and arrange for follow-up (Jay et al., 2010a). ‘Physicians' use of the 5As is associated with higher odds of patient motivation to lose weight, intention to eat healthier, and intention to exercise’ (Jay et al., 2010b). ‘The 5 As, developed for smoking cessation, can be adapted for obesity counseling. The 5 As are appealing, as they are rooted in behaviour change theory and can be implemented in busy practice settings’ (Vallis et al., 2013).
	Motivational interviewing (MI)	Principles for discussing change behaviour. Focus on patient’s thoughts and perspectives, with an aim to overcoming ambivalence and moving through the change cycle.	‘Motivational interviewing (MI) is one approach to patient-centered communication that addresses behavior change. MI may be used by health care providers to explore and resolve ambivalence regarding behavioral change. When physicians use MI when speaking with patients, they seek to elicit patients’ own reasons for change, act as partners, are supportive, explore their patients’ concerns, and convey that patients are the drivers of their own change process’ (Bravender et al., 2013). ‘…use of MI consistent techniques was associated with improvement in patient confidence to improve nutrition as well as a modest increase in patient reported exercise level’ (Cox et al., 2011). ‘The principles of MI, including providing empathy, collaborating with clients, and supporting client autonomy, are consistent with the elements of patient-centered care and consensus recommendations for working with clients from different cultures in obesity treatment’ (Carcone et al., 2013). ‘The technique of motivational interviewing (MI) can also effectively promote weight loss. Patients’ whose primary care providers employed MI consistent techniques during counseling demonstrated greater confidence to change their diet’ (Gudzune et al., 2012). ‘MI is patient-centered, not doctor-centered. This means that the physician listens to the patient’s perspective on how the problem affects daily life and seeks to understand the patient’s point of view without judging or criticising the behavior. The goal of MI is to elicit the patient’s motivation to change and to encourage the patient to take responsibility for his/her behavior’ (Schwartz, 2010).
	4 E’s	Similar framework to 5A’s: engage, empathise educate, enlist General communication technique, not specific for weight loss	The 4E model was chosen to teach anaesthetic trainees communication skills because ‘it is based on literature that reflects both primary care and procedural settings… it has been widely and successfully used in brief workshops with physicians…’ (Eggly et al., 2003)
	Written materials	Potential to address inconsistencies in weight loss advice with written materials for patients to receive	‘Weight management activities in primary care are limited and inconsistent, with GPs reluctant to raise the subject of weight, and many lacking confidence in existing treatments. [Standardised written material] intervention addresses these issues, offering a simple, low-cost, treatment that can be delivered by primary care staff without special expertise’ (Beeken et al., 2012).
Language and communication	Empathetic	Expression of empathy is related to positive feedback from patients receiving weight loss advice	‘…participants indicated that they were more likely to have favourable weight-related interactions with physicians who possessed certain qualities, such as being empathetic, sensitive, respectful, trustworthy, compassionate, nonjudgmental, encouraging, honest, and comforting’ (Chugh et al., 2013). ‘…despite the overall paucity of expression of empathy, its presence was associated with improvement in Fat and Fiber scores as well as trends towards improvement in motivation scores and weight loss attempts’ (Cox et al., 2011).
	Patient-centred (specific)	Advice to patients is more meaningful if personalised and not generalised weight loss advice	‘…participants expressed a desire for specific advice and personalised weight management plans… when women received generalised and nonspecific weight loss advice from their physician, they equated this with lack of concern, attention and support’ (Chugh et al., 2013). ;Providers should make dietary and physical activity advice in pregnancy more clear and individualised and offer such guidance multiple times throughout pregnancy’ (Ferrari et al., 2013). ‘Physicians may be able to improve care for their obese patients by focusing discussions on specific details of diet and physical activity behaviors, and by clarifying that patients perceive weight-related information has been shared’ (Greiner et al., 2008). ‘A clinician engaging in patient-centered, shared decision making may be most likely to tailor specific behavioral recommendations for patients to consider, adjust the amount of information conveyed, and arrange referrals to appropriate external resources’ (Greiner et al., 2008). ‘As with all health behavior change initiatives in health care, general statements from a physician may be less effective than stage assessment, specific advice or assistance, tailored counseling, and resource coordination’ (Greiner et al., 2008). ‘…the patient-centeredness of the physician was strongly associated with patient intentions… suggests that how counseling skills are delivered matters and that quality of the physician/patient relationship may influence the patients' commitment to behavior change’ (Jay et al., 2010b).
	Specific terminology	Specific terminology to use Specific terminology to avoid Acknowledgement of patients excess weight	‘Many people thought “overweight” or “heavy” would be the most acceptable way for someone to describe their weight status… “Large”, “High BMI”, “Unhealthy weight” and “Excessive weight” were also endorsed as acceptable terms’ (Gray et al., 2011). ‘Reactions to the terms “obese”, “fat” and “excessive fat” were usually adverse’ (Gray et al., 2011). ‘“Unhealthy BMI”, “High BMI” and “Unhealthily High Body Weight” were often felt to be good terms to motivate weight loss: they were seen as professional and providing a clear definition of the problem’ (Gray et al., 2011). ‘Patients rated “weight” as the most desirable term for their physician to use to describe overweight or obesity, whereas “fatness” was the least desirable term rated by patients’ (Dutton et al., 2010). ‘…twice as many parents preferred the term *gaining too much weight* compared with the term *overweight*. Further, the authors reported that if parents perceived weight-related terms as hurtful or judgmental during healthcare communications, such perceptions negatively influenced even the most well-intentioned intervention’ (Farnesi et al., 2012). ‘The terms “fatness” and “obesity” have negative connotations and elicit negative reactions in patients; whereas “weight”, “overweight” and “Body Mass Index” were judged favourably, with “weight” being the most desirable term’ (Mikhailovich & Morrison, 2007). ‘…the terms “fat” and “fatness” are the least preferred terms. The words “obese” and “obesity” have also been found to arouse negative responses’ (Gordon & Black, 2016) ‘…patient reports of being told by a physician that they were overweight were associated with more realistic perceptions of the patients’ own weight, desire to lose weight, and recent attempts to lose weight’ (Post et al., 2011).
Specific recommendations	Training for physicians	Training for physicians may be of benefit in overcoming barriers to weight loss conversations	‘Non primary-care specialties may need to tailor current physician-patient communication models to their setting in order to train residents in interpersonal and communication skills’ (Eggly et al., 2003). Ashby et. al. stated that education and confidence in knowledge had a positive impact on likelihood to provide weight loss advice, therefore targeted education programs to provide current information could provide positive behaviour change (Ashby et al., 2012).
	Clear referral pathways	Clear referral pathways should be available for management of overweight/obesity	‘Office-based approaches to obesity management remain extremely challenging. Early recognition of overweight and obesity and communication to patients about the realities of their weight is an important initial step to successful behavior change. Implementing or referring patients for intensive weight loss interventions may be effective for some patients’ (Baron, 2011). ‘Formalisation of referral pathways and follow up is currently lacking and could assist rural GPs in helping their patients to exercise and lose weight’ (Eley & Eley, 2009). ‘Patients should be assisted in identifying and seeking out credible weight-management resources and be referred to appropriate providers for management (i.e. emphasizing an interdisciplinary approach). Arranging follow-up is important so that the support of the physician recommendations can continue’ (Vallis et al., 2013).
	Specific consultation suggestions	Some specific consultation suggestions regarding perioperative overweight/obesity conversations: - Acknowledgement of overweight/obesity initially - Use of checklists for practitioners - Patients to complete short form regarding diet and exercise prior to consultation - Discussion of increased risks of obesity in perioperative period - Limit amount of information conveyed so as to not overwhelm patient - Brief interventions have significant impact on smoking cessation, potential for similar impact in weight loss	‘Health care providers should acknowledge their patient’s excess body weight as a first step in counselling patients…’ (Ahn et al., 2012) ‘Use of chart stickers and checklist forms to remind practitioner—also patients can complete short form regarding diet and exercise while waiting’ (Ahn et al., 2012). Suggestion for candid discussion of increased risks in the perioperative period with obese patients including the suggestion that surgery is deferred to allow further time for weight loss (Astin & Hardy, 2015). Anaesthetists can overload patients with more information than they can process in the pre-anaesthetic visit: ‘At baseline, an average individual can recall approximately seven “chunks” of new information. Against this backdrop, we observed an extreme tendency toward information overload by health care providers, coupled with a failure to use memory-enhancing techniques’ (Sandberg et al., 2008). ‘Even when doctors provide brief simple advice about quitting smoking this increases the likelihood that someone who smokes will successfully quit and remain a nonsmoker 12 months later’ (Stead et al., 2013).

An additional theme relating to obesity-related conversations in special groups such as paediatrics, obstetrics and different cultures, ages or gender was explored but not found to add to the specific study question. These data are not reported.

## Discussion

### Barriers to conversations

The most common theme from the literature concerned the perceived existence of multiple barriers to healthcare professionals having conversations with patients about their obesity. This theme was further stratified into several specific barriers, with one paper ranking the barriers according to physician feedback (Table 4) (Huang et al., 2004). The overarching premises surrounding this theme related to either lack of skills, training and time, and a perceived futility of such conversations. A recent paper described the effectiveness of a 7.5-h communication-training program for improving physicians’ communication self-efficacy, attitudes and behaviours (Saslaw et al., 2017). This paper stated that there are benefits of brief communication skills training to both physician and patient satisfaction (Saslaw et al., 2017). Despite the recognised barrier of time constraints, the evidence for success of brief interventions in smoking cessation is encouraging for weight loss (Stead et al., 2013). The premise of the efficacy of the brief weight loss intervention coincides with the concept of the ‘teachable moment’, which is discussed further in specific recommendations below.

**Table 4:** Ranked physician-reported barriers to weight loss conversations *(Huang et al.,*^2004^*)*

Ranked physician-reported barriers to weight loss conversations:	
1. Pessimism about patient's desire and ability to lose weight	
2. Pessimism about effectiveness of weight loss counselling	
3. Lack of comprehensive obesity management resources (e.g. weight loss clinic)	
4. Insufficient time due to high patient volume	
5. Underuse of dieticians or lack of experience working with dieticians	
6. Lack of skills in providing brief counselling	
7. Insufficient knowledge of best clinical practices	

Although none of the reviewed articles related directly to preoperative anaesthesia consultations, these barriers appeared to be relevant enough to be applicable and potentially useful to this situation.

### Communication tools

Specific communication tools were examined or explained in many of the papers. These included specific guidelines such as the 5A’s and the 4E’s frameworks that provide a structured approach, as well as more broad reference to communication methods such as use of motivational interviewing (MI) and use of written materials given to patients. Description of the use of these tools mainly emerged from primary care, with a focus on evidence-based practice in overweight/obesity conversations. The 5A’s and MI were the most commonly reported communication tools: these have previously been used with success in addressing smoking cessation (Fiore et al., 2008; Pollak et al., 2010). The difference between smoking cessation and dietary advice was highlighted by Phillips et al: ‘Success for smoking is measured as an absolute (smoking cessation), but success for healthy eating traverses along a continuum, measured by various factors (such as weight loss, and reduced cholesterol level)’ (Phillips et al., 2012). Likewise, Jebb et al. noted that unlike smoking cessation, campaign messages of ‘Stop Smoking’, the messages to obese patients are much more complex as cessation of eating is not an appropriate intervention (Jebb et al., 2003).

Despite the differences between weight management and smoking cessation, the shared underlying concept of these tools is to move patients through the change cycle towards self-motivated behaviour change (Jay et al., 2010a; Schwartz, 2010). While both MI and the 5A’s are described as effective tools in weight loss conversations (Gudzune et al., 2012), the simplicity of the 5A’s may make it more appropriate for preoperative use. The 5A’s was initially designed for smoking cessation (Manley et al., 1992) but there is clinical evidence for its use in weight management (Jay et al., 2010a; Alexander et al., 2011). A modified version of the 5A’s framework has been proposed for Canadian family physicians in the primary care setting (Vallis et al., 2013). The primary aim of the recommendations is for the physician to initiate sensitive conversations with the patient, to help empower patients to pursue their own weight loss goals and endeavours (Vallis et al., 2013). The 5A’s is also utilised in the Australian Government Department of Health National Health and Medical Research Council guidelines for management of overweight/obesity in primary care (NHMRC, 2013). These examples demonstrate the potential simplicity that could be utilised in the preoperative consultation. A communication tool that is relatively straightforward to teach and learn will also be important for the design and implementation of physician training programmes. A potential challenge to the implementation of the 5A’s in the preoperative period is that ‘assist’ and ‘arrange’ are the most important aspects (Alexander et al., 2011), but may be difficult to accomplish with the limited time frame and competing priorities of the preoperative consultation (Kopp & Shafer, 2000). A clear referral structure would help anaesthetists provide the most effective weight loss guidance for patients, and could include established pathways for referral to an exercise physiologist, dietician, psychologist, dedicated public health weight loss programmes or back to the primary care physician.

### Language and communication

Several papers made reference to the specific language used in weight loss conversations. Some papers considered the broader concept of empathy, noting that patients wanted their physicians to be empathetic when holding weight loss conversations (Chugh et al., 2013; Farnesi et al., 2012). In terms of more specific communication language, papers identified particular terminology to use, and terminology to avoid, when describing the condition of overweight/obesity with patients (Dutton et al., 2010; Gray et al., 2011). Preferred terminology was partially dependent on patient demographics (Farnesi et al., 2012; Gray et al., 2011; Raaff et al., 2014), making it difficult to provide broad, definitive instructions for future guidelines. However, some terms were noted in multiple papers to be undesirable when discussing overweight/obesity with patients, such as ‘fat’, ‘fatness’, ‘obese’ and ‘obesity’ (Dutton et al., 2010; Mikhailovich & Morrison, 2007; Gordon & Black, 2016; Tailor & Ogden, 2009). Multiple papers reported ‘weight’ to be the most favourable term (Dutton et al., 2010; Mikhailovich & Morrison, 2007). Communication should also be patient-centred, and specifically tailored to the individual (Chugh et al., 2013; Greiner et al., 2008). Patients derive less meaning from generalised weight loss information (Chugh et al., 2013; Greiner et al., 2008). A simple acknowledgement of a patient’s overweight status has been associated with increased desire and attempts to lose weight (Post et al., 2011). This holds parallels with the so-called teachable moment of smoking cessation (Gritz et al., 2006), and may hold promise for discussions about weight in the preoperative period. The ‘teachable moment’ relates to the interaction between patient and healthcare provider at a time when a patient may be particularly receptive to heath information. This can occur in situations that involve increased perceptions of risk, adverse outcomes, emotional situations or that involve a re-definition of self (McBride et al., 2008). Examples include times of a diagnosis of cancer, pregnancy, an upcoming operation or major illness. This concept has been widely used in smoking cessation and has been described in the management of obesity (Phelan, 2010). Patients expect to receive health information and counselling whenever they interact with health care professionals, and a lack of this may be perceived as an affirmation of poor health practices (Pool et al., 2014). The perioperative period represents an opportunity to utilise this expectation as a ‘teachable moment’ (Wynter-Blyth & Moorthy, 2017). Assessment prior to an operation could be considered to be a time of ‘openness’ to healthcare information from several perspectives, and thus the preoperative anaesthetic assessment could be utilised to present obesity management information in an effective manner.

### Specific recommendations

The articles gave suggestions on how to overcome the identified barriers and incorporate effective language and tools in weight loss conversations. In recognising that most anaesthetists are not trained in providing detailed overweight/obesity advice and are uncertain and even uncomfortable in doing so, the establishment of well-defined referral pathways to dieticians or other specialists was an important recommendation (Vallis et al., 2013; Eley & Eley, 2009; Baron, 2011). This is particularly relevant for anaesthetists as they do not have an ongoing relationship with patients over time. Multiple articles highlighted the need for specific training of physicians, primarily around conversational techniques (Eggly et al., 2003; Ashby et al., 2012).

We found several recommendations that may be usefully considered as guidelines for preoperative weight loss discussions. These include acknowledgement of overweight/obesity in the initial consultation (Ahn et al., 2012), use of specific tools and checklists to open the discussion and assess the position of the patient on the change cycle (5As, MI) (Ahn et al., 2012), patients completing a short form on diet and exercise prior to the consultation (Ahn et al., 2012) and discussion of increased risks of obesity with reference to the specific planned surgery (Astin & Hardy, 2015). There should also be a limit on the overall amount of information provided to the patient, focussing on key messages, as patients have limited capacity to integrate a large amount of information in the preoperative setting (Sandberg et al., 2008). Training anaesthetists in these specific communication skills will be important. The support of pre-arranged consultation and referral pathways is particularly relevant in an anaesthesia preadmission setting. With clear referral pathways, patients can be followed through the change cycle by their primary care physician or allied health professional, with the goal of long-term, sustained weight loss.

## Limitations

There are several limitations to this review. There was a lack of directly pertinent research to the field of anaesthesia, which made compilation of practice recommendations for the field of anaesthesia challenging. However, this is also indicative that this is truly novel research, and the first work to suggest guidance for practice, and these suggestions can now be tested or clinically implemented for future investigations. A further limitation was the heterogenous nature of the sources utilised for the narrative review. This did enable a broad range of sources from which to draw recommendations, which may not have been possible if the source material was more homogenous. The review was also initially planned as a systematic review, which would have provided a high level of evidence than the performed narrative review. There was a lack of high-quality quantitative studies to include; therefore, the majority of the information incorporated in this review is qualitative in nature.

To summarise the main messages from this narrative review, we propose the following practice points that may be helpful for anaesthetists in preoperative consultations with patients with obesity.

### Practice points to consider

- Train anaesthetists and other perioperative medicine practitioners in obesity specific communication skills, including use of empathetic language
- Patients can receive information about the effects of obesity on anaesthesia and surgery, and complete a short form on diet and exercise, prior to their preoperative review.
- Start the conversation by acknowledging patient obesity using an empathetic approach: ‘I am concerned about your unhealthy weight’ or ‘Are you OK if we talk about your weight?’
- Use a formal communication tool such as the 5A’s to help structure the discussion and assess the position of the patient on the change cycle (Table 5).
- Discuss the increased risks of obesity with reference to the specific patient and planned surgery.
- Communication should be patient-centred and specifically tailored to the individual.
- Have pre-arranged consultation and referral pathways for ongoing care.
- Provide written materials and/or web-links for online support and reliable information for patients to take away.

**Table 5:** Example of the 5A’s approach to weight management

Ask	- Permission to discuss weight - Measure body mass index - About comorbidities - About other factors related to health risk, e.g. smoking, alcohol, exercise
Assess	- How do you feel about your weight at the moment? - Do you feel ready to think about losing some weight/improving your fitness/health?
Advise	- ‘The best thing you can do for your health is to lose weight’ - Promote the benefits of a healthy lifestyle - Explain the benefits of weight loss for the specific surgery
Assist	- The particular approach to follow will depend on results of the ‘assess’ phase, i.e. how ready the patient is to act on their obesity: patients may be ready, unsure, or not ready to change - Help patient to identify and plan to address the barriers to weight loss that are relevant to them - Help patient to start to develop a weight management plan
Arrange	- Referral and follow-up as required (e.g. to a primary care physician, dietician, exercise physiologist or psychologist) to oversee long-term weight management

Modified from Vallis et al. (Vallis et al., 2013) and Australian Government clinical practice guidelines for managing obesity (NHMRC, 2013)

## Conclusions

There is no existing literature to directly guide anaesthetists in conducting effective preoperative communications with patients who have obesity. Some potential guidelines and considerations for having these discussions in an effective and empathetic manner are proposed. Further research is required to provide evidence-based recommendations for this increasingly important issue.

## Data Availability

Not applicable.
